# Spinal epidural hematoma following placement of a thoracic spinal cord stimulator

**DOI:** 10.1016/j.inpm.2022.100117

**Published:** 2022-07-08

**Authors:** Vishwant Tatagari, David Simons

**Affiliations:** Department of Anesthesia, UPMC Lititz, 1500 Highlands Drive, Lititz, PA, 17543, United States

Dear Editor,

Spinal cord stimulation is a useful modality to treat many non-malignant causes of chronic pain. While extremely rare, spinal epidural bleeding and hematoma are a known complication of spinal cord stimulator (SCS) lead placement. We present a case of a spinal epidural hematoma that was identified shortly after implantation of a permanent thoracic spinal cord stimulator.

A 71-year-old female with a past medical history of lumbar radiculopathy, chronic pain disorder, Parkinson's disease, asthma, gastroesophageal reflux disease (GERD), hypothyroidism, migraines, anxiety, and depression presented to the emergency department via ambulance at roughly 1800 after an unwitnessed fall at home. She stated that she got up out of a chair to go to the bathroom and her legs felt weak and “gave out on her.” Patient reported a history of lumbar radiculopathy with chronic back and leg pain. She typically ambulates with a walker. Her Parkinson's disease was well-controlled on carbidopa/levodopa. Other home medications included albuterol, bupropion, aripiprazole, cyclobenzaprine, clonazepam, duloxetine, gabapentin, levothyroxine, sumatriptan and pantoprazole. She denied use of NSAIDs or herbal supplements. She had a thoracic spinal cord stimulator placement with left-sided T9 and T10 hemilaminectomies the morning of the fall and was discharged home the same day as the procedure. In the emergency department she reported left leg weakness and paresthesia of the right thigh. She also reported left shoulder pain that resolved with oral acetaminophen.

On physical exam lower extremity sensation was intact bilaterally with 5/5 strength of the right lower extremity but 3/5 strength with left plantarflexion and 4/5 with left dorsiflexion. Left hip flexion was found to be decreased. Additionally, she was unable to actively lift her left leg. She denied back pain at the surgical incision site and the site was found to be clean, dry, and intact. The patient also presented with urinary retention. She was unable to void in the ER. Bladder scan displayed 870 ​cc of urine in the bladder for which a Foley catheter was placed. Laboratory studies were unremarkable and vital signs were within normal limits.

Neurosurgery was consulted by phone. The neurosurgeon who performed the prior procedure recommended turning off the spinal cord stimulator. However, the patient had left the remote at home and did not have family nearby who were able to bring it. No imaging studies of the spine were recommended or performed at the time. The patient spent the night in the ER and in the morning a representative from the medical device company was able to bring a remote to turn the device off. Due to the delay in diagnoses a non-contrast CT of the thoracic spine was completed to save time as opposed to an MRI, even though it is the gold standard imaging modality. The CT revealed that the epidural leads entered at T10-11 with the tip of the paddle electrode located at the inferior portion of T8 (Fig. 1). The leads were located more centrally within the spinal canal causing some central stenosis, potentially due to a posterior epidural hematoma.

**Fig. 1:**
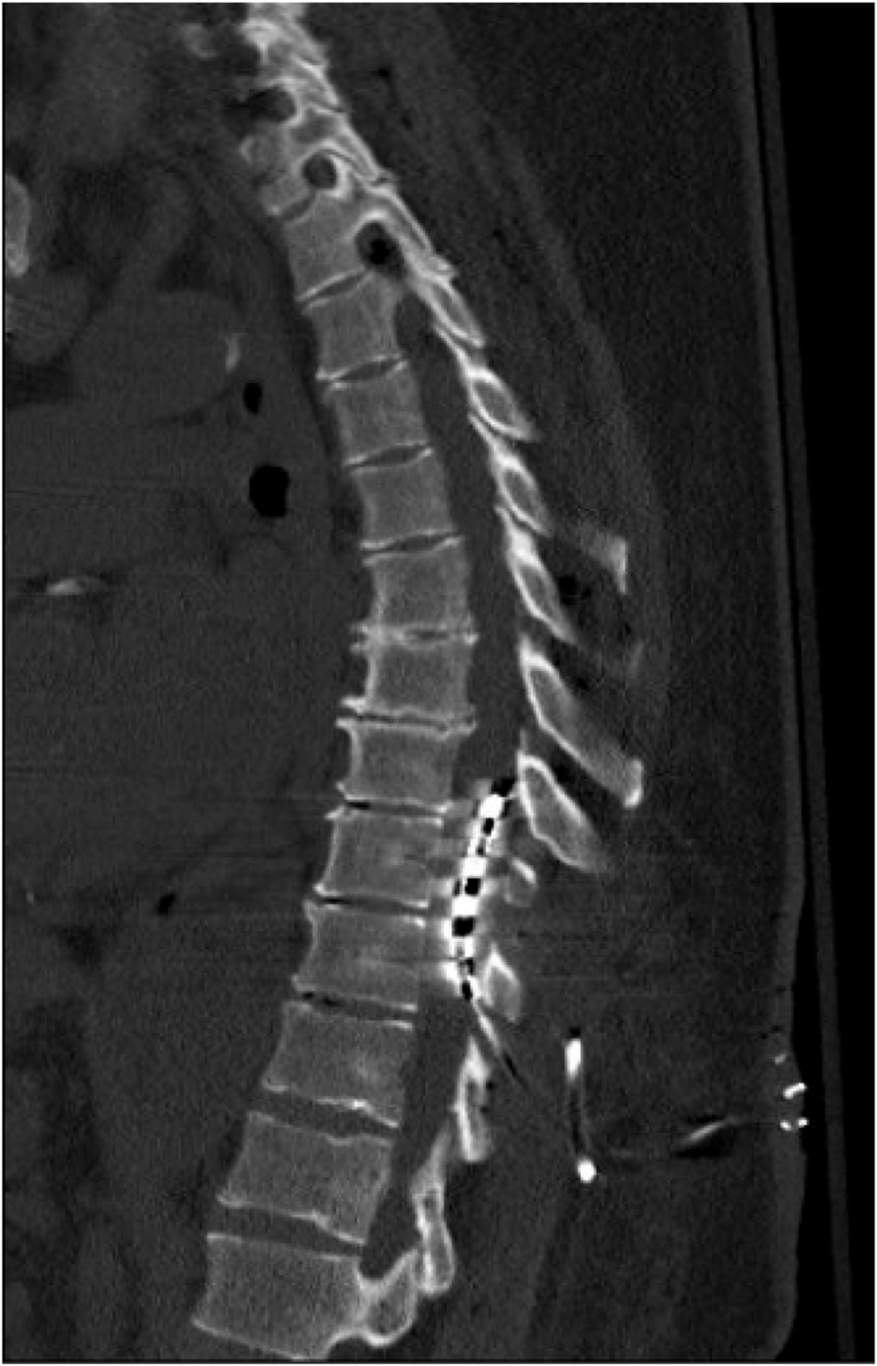
Spinal cord stimulator leads entering at T10-11 with the tip of the paddle electrode at the inferior portion of T8.

Shortly after the device was turned off and the CT was obtained the neurosurgeon came to evaluate the patient in the ER. At this time strength was found to be 5/5 throughout in the right lower extremity and 5/5 in the left lower extremity apart from left hip flexion which was 4/5 at baseline. The patient did report the weakness and paresthesia resolved after the device was turned off. However, she was still weak on her feet and with ambulation. Although the imaging findings were questionable, after discussing potential options with the patient she agreed to undergo urgent removal of the device.

After general anesthesia was found the be adequate the patient was placed in the prone position and draped in sterile fashion. The prior midline incision was incised with full dissection exposing both T9 and T10 lamina. Upon full exposure there appeared to be a small epidural hematoma at the left-sided T9 hemilaminectomy site. There appeared to be no compression. A full laminectomy was performed at T10 followed by removal of the epidural leads. Following this a T9 laminectomy was performed. At this point the spinal epidural hematoma was fully visualized. It extended up to the inferior portion of T8. It was left-sided and non-compressive. The hematoma was evacuated and a T8 left hemilaminectomy was performed to ensure adequate evacuation of the blood clot.

After this the left gluteal incision was incised and the generator was removed from its pocket along with the stimulator leads. Both incisions were irrigated, and muscle, fascia, and skin were closed with sterile sutures. The patient was then extubated and taken to the recovery room in stable condition.

The following morning the patient's Foley catheter was removed, and she was able to adequately void. The patient began physical therapy and reported improvement in the leg weakness and denied paresthesia. On post-operative day 1 lower extremity strength was found to be 5/5 bilaterally. She utilized oral analgesics for pain control. The patient continued to progress with physical therapy and was discharged to a skilled nursing facility on post-operative day 3.

A 2014 retrospective analysis of epidural catheterizations found 0.0002% (1:7200) incidence of spinal cord hematoma [1]. A 2011 retrospective analysis of neurologic injury following paddle type SCS implantation reported an incidence of 0.6% in 44,587 cases [2]. Epidural hematoma was reported in 83 (0.19%) of these cases [2]. A 2016 retrospective analysis by Petraglia et al. found a combined epidural hematoma incidence of 0.70% in 8326 patients undergoing thoracic SCS placement with either percutaneous or paddle leads [3]. A retrospective study by Moufarrij reported epidural hematoma formation in 4 of 154 (2.60%) of patients undergoing paddle SCS placement. However, in this study all stimulators were placed by the same surgeon. While all these studies differ in their incidence reporting, it can be concluded that spinal epidural hematoma formation after SCS placement is incredibly rare.

A spinal epidural hematoma can occur spontaneously or be related to coagulopathies, vascular malformations, tumors, trauma, and spinal procedures [4]. Other risk factors include age (greater than 50 years old), male sex, and multi-level laminectomies [4]. It is theorized that one mechanism of spinal epidural hematoma after epidural catheterization is due to hemorrhage of the internal vertebral venous plexus [4]. This plexus is a network of interconnecting veins located in the epidural space with two longitudinal channels in the anterolateral epidural space and two segmental channels in the posterolateral epidural space. The posterior channels are located more laterally in the cervical and lumbar regions. Lead migration may result in trauma to these vessels resulting in a spinal epidural hematoma [4].

MRI is the gold standard imaging modality for spinal epidural hematomas. If an MRI cannot be performed or is not available a non-contrast CT or CT myelogram may be performed. However, a CT may give a false-negative if the hematoma is isodense to the thecal sac or spinal cord [5]. Additionally, spinal CT in the thoracic spine may be non-diagnostic as there may be poor resolution due to high contrast between the lung parenchyma vertebral bone [5]. In our case the CT of the thoracic spine was concerning but not diagnostic for a spinal epidural hematoma. A CT myelogram involves injecting dye into the spinal cord. This is invasive, time consuming and may worsen neurological symptoms.

While rare, it is prudent to recognize signs and symptoms of neurologic injury following SCS implantation. In our case, the symptoms were initially thought to be related to the stimulator function itself as opposed to a complication from implantation leading to a delay in imaging and diagnosis. There are reports suggesting that spinal epidural hematomas can be managed conservatively in patients with mild symptoms and returning neurological function [6]. However, there is no data on if patients with implanted devices can undergo conservative treatment. This patient was treated within 24 hours of presentation. Even though the patient did have improvement of symptoms after turning off the stimulator, it is not clear what the outcome would have been had any further delay in surgical treatment had occurred, or if conservative management with observation and repeat imaging studies would have led to the same outcome since this patient had an implanted device. If a spinal epidural hematoma is suspected after SCS implantation urgent evaluation with a thorough physical exam and imaging studies followed by surgical evacuation of the hematoma should be undertaken to prevent permanent injury.

## Funding

This research did not receive any specific grant from funding agencies in the public, commercial, or not-for-profit sectors.

## Declaration of competing interest

The authors declare that they have no known competing financial interests or personal relationships that could have appeared to influence the work reported in this paper.
