# Supplementary material for: Spinal epidural hematoma following placement of a thoracic spinal cord stimulator
Source: Interv Pain Med. 2022 Jul 8;1(3):100117. doi: 10.1016/j.inpm.2022.100117 (PMC11372968; doi:10.1016/j.inpm.2022.100117)
Supplement: Multimedia component 1 [file mmc1.docx]

**UPMC Harrisburg**

May 18, 2022

Vishwant Tatagari, MD

Principal Investigator

Re: Case study

Dear Dr. Tatagari,

Thank you for your interest/intent to author/publish a case study of a female patient who presented with lower extremity weakness and urinary retention after spinal cord stimulator placement. This research is exempt under 45 CFR 46.101.

If we can be of any further assistance to you, please contact the IRB Office at (717) 231-8394 at your convenience.

Sincerely yours,

Thomas Pineo, DO

Thomas Pineo, DO

Chairman

UPMC Central PA Region

Institutional Review Board

TP:nf

**UPMC Central PA Region**

**Institutional Review Board**

**Thomas Pineo, D.O.**

IRB Chairman

111 S. Front Street

Harrisburg, PA 17101

PO Box 8700

Harrisburg, PA 17105-8700

717-231-8394

Inquiries:

Nancy Fisher

IRB Coordinator

**fishernl2@upmc.edu**

DHHS OHRP Registration:

IORG0001079

IRB000001476

FWA00001055
